# Supplementary material for: Splice-Junction-Based Mapping of Alternative Isoforms in the Human Proteome
Source: Cell Rep. Author manuscript; Available in PMC 2020 Jan 15. (PMC6961840; doi:10.1016/j.celrep.2019.11.026)
Supplement: 2 [file NIHMS1546469-supplement-2.zip › DF1/PXD006675/RightVentricle/RightVentricle_52_TTN_ETPPVEVPGGEK.pdf]

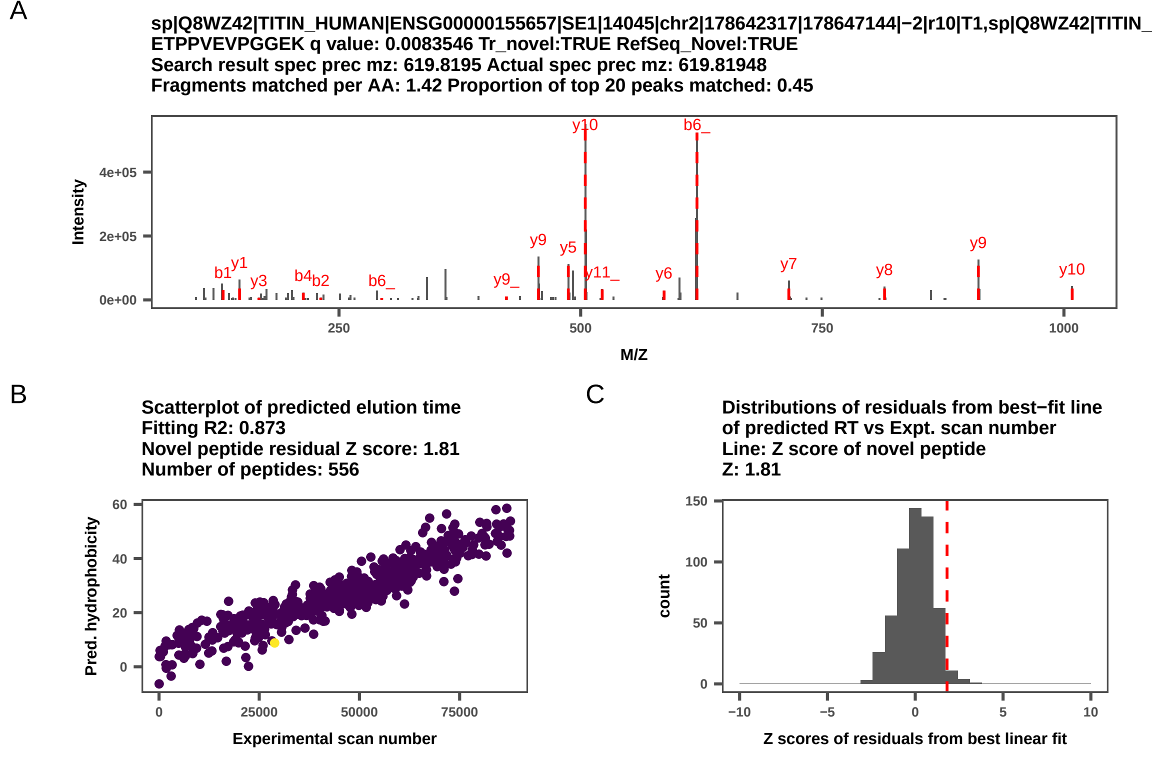
A
sp|Q8WZ42|TITIN_HUMAN|ENSG00000155657|SE1|14045|chr2|178642317|178647144|−2|r10|T1,sp|Q8WZ42|TITIN_
ETPPVEVPGGEK q value: 0.0083546 Tr_novel:TRUE RefSeq_Novel:TRUE
Search result spec prec mz: 619.8195 Actual spec prec mz: 619.81948
Fragments matched per AA: 1.42 Proportion of top 20 peaks matched: 0.45
Intensity
4e+05
2e+05
0e+00
y10
b6_
y9
y5
y9
b1
y1
y3
b4
b2
b6_
y9_
y11_
y6
y7
y8
y10
250
500
750
1000
M/Z
B
Scatterplot of predicted elution time
Fitting R2: 0.873
Novel peptide residual Z score: 1.81
Number of peptides: 556
Pred. hydrophobicity
60
40
20
0
0
25000
50000
75000
Experimental scan number
C
Distributions of residuals from best−fit line
of predicted RT vs Expt. scan number
Line: Z score of novel peptide
Z: 1.81
count
150
100
50
0
−10
−5
0
5
10
Z scores of residuals from best linear fit
